# Utilization of interdisciplinary in-hospital early rehabilitation in COVID-19 patients - a multicenter cohort study in the National Pandemic Cohort Network (NAPKON) in Germany

**DOI:** 10.1371/journal.pone.0334941

**Published:** 2025-10-31

**Authors:** Max E. Liebl, Anett Reisshauer, Dana Loudovici-Krug, Philipp Baumbach, Katharina S. Appel, Sabine Blaschke, Johanna Erber, Ilka Grewe, Marina Hagen, Ekaterina Heim, Sina M. Pütz, Kristin Lehnert, Patrick Meybohm, Olga Miljukov, Milena Milovanovic, Susana M. Nunes de Miranda, Christoph Römmele, Phil-Robin Tepasse, Jörg J. Vehreschild, Norman Weinert, Martin Weigl, Julia Wendel, Christina Lemhöfer

**Affiliations:** 1 Department of Physical and Rehabilitation Medicine, Charité – Universitätsmedizin Berlin, corporate member of Freie Universität Berlin and Humboldt Universität zu Berlin, Berlin, Germany; 2 University Hospital Jena, Institute of Physical and Rehabilitation Medicine, Friedrich-Schiller University Jena, Jena, Germany; 3 Department for Anesthesiology and Intensive Care Medicine, University Hospital Jena, Friedrich-Schiller University Jena, Jena, Germany; 4 Faculty of Medicine, Goethe University Frankfurt, Institute for Digital Medicine and Clinical Data Science, Frankfurt, Germany; 5 Central Emergency Department, University Medical Center Göttingen, Göttingen, Germany; 6 TUM School of Medicine and Health – Clinical Department for Internal Medicine II, TUM University Hospital, Technical University of Munich, Munich, Germany; 7 I. Department of Medicine, University Medical Center Hamburg-Eppendorf, Institute for Infection Research and Vaccine Development, Hamburg, Germany; 8 Department II of Internal Medicine, Hematology/Oncology, Goethe University, Frankfurt, Germany; 9 Trusted Third Party of the University Medicine Greifswald, Greifswald, Germany; 10 Faculty of Medicine and University Hospital Cologne, Department I of Internal Medicine, University of Cologne, Center for Integrated Oncology Aachen Bonn Cologne Duesseldorf, Cologne, Germany; 11 DZHK (German Center for Cardiovascular Research), University Medicine Greifswald, Greifswald, Germany; 12 Department of Internal Medicine B, University Medicine Greifswald, Greifswald, Germany; 13 Department of Anaesthesiology, University Hospital Würzburg, Intensive Care, Emergency and Pain Medicine, Würzburg, Germany; 14 University of Würzburg, Institute for Clinical Epidemiology and Biometry, Würzburg, Germany; 15 University Hospital Würzburg, Institute for Medical Data Science, Würzburg, Germany; 16 Malteser Krankenhaus St. Franziskus Hospital, Medical Clinic I, Flensburg, Germany; 17 Internal Medicine III – Gastroenterology and Infectious Diseases, University Hospital of Augsburg, Augsburg, Germany; 18 Department of Medicine B for Gastroenterology, University Hospital Münster, Hepatology, Endocrinology and Clinical Infectiology, Münster, Germany; 19 German Center for Infection Research (DZIF), Partner-Site Cologne-Bonn, Cologne, Germany; 20 Department of Medical Informatics, University Medical Center Göttingen, Göttingen, Germany; 21 Department of Orthopaedics and Trauma Surgery, Musculoskeletal University Center Munich (MUM), University Hospital, LMU Munich, Germany; MountainView Hospital, UNITED STATES OF AMERICA

## Abstract

**Background:**

Early rehabilitation in acute hospitals aims to prevent immobilization-related complications and improve the functional capacity of patients with severe or critical illness. Early rehabilitation can be a useful concept to improve functioning in COVID-19 patients. However, literature concerning early in-hospital rehabilitation in COVID-19 patients is scarce.

**Aim:**

To analyze the utilization of in-hospital interdisciplinary early rehabilitation (IER) in COVID-19 patients and characterize the sample of IER patients.

**Design:**

Prospective cohort study.

**Setting:**

Hospitalized COVID-19 patient cases.

**Population:**

This study used data from the National Pandemic Cohort Network (NAPKON) in Germany.

**Methods:**

IER utilization rates were retrieved. Demographic and clinical data from hospitalized COVID-19 patients who had received IER during the course of their treatment were evaluated.

**Results:**

Out of the 2,644 patients in the Cross-Sectoral Platform (German abbreviation: SUEP) cohort, 0.79% [95% CI: 0.51% to 1.22%] received IER during their stay in an acute care hospital. Among the subgroup of patients who had previously been treated in intensive care, 2.13% [95% CI: 1.16% to 3.63%] received IER. The most common comorbidities were cardiovascular diseases (66.7%) and neurological/psychiatric diseases (36.1%). The small sample size limited further analyses.

**Conclusion:**

The low rate of early rehabilitation in acute hospitals for COVID-19 patients indicates an unmet need, particularly in severe cases. Structural changes in the health system are needed to close this gap. The WHO and the German Medical Council have recently acknowledged the necessity of early in-hospital rehabilitation and have issued a call for its implementation in acute hospitals.

## Introduction

Early rehabilitation (ER) is a rehabilitation intervention that begins at the earliest possible point in the course of the treatment of disease or trauma [1]. German social law defines ER as part of hospital treatment and therefore distinguishes it from the rehabilitation sector [1]. In addition to geriatric ER and neurological/neurosurgical ER, the interdisciplinary early rehabilitation (IER) is also available in acute inpatient settings in Germany. In contrast to its legal definition as an integrative part of hospital treatment, ER is often carried out in specialized hospitals to which patients are transferred from the acute hospital. However, some centers, predominantly university hospitals and maximum care providers, offer IER services integrated in the acute hospital itself. This is particularly suitable for patients who still require the diagnostic or therapeutic infrastructure of an acute hospital [1].

Data on the clinical effects of ER in general are sparse, but there are some, particularly diagnosis-specific studies. Following stroke or traumatic brain injury, there is evidence that ER after acute respiratory distress syndrome (ARDS) can result in improved functional outcomes. The prevention of immobilization-related complications has also been demonstrated [2–4]. After sepsis, ER has been shown to reduce mortality compared to later rehabilitation [5]. Observational studies show high functional gains with regard to independence and mobility both in an interdisciplinary cohort and after polytrauma, and also after COVID-19 [6–8]. After oncological therapy, ER can be a strategy to avoid transfer to nursing homes [9]. ER not only includes intensified physiotherapeutic treatment, but also an overall interdisciplinary concept involving various professional groups in a multi-professional team with a rehabilitation specialist as team leader. In Germany, structural requirements for IER are specified in code 8–559 in the operations and procedures code (German abbreviation: OPS) of the German diagnosis-related groups system (G-DRG) [10]. Table 1 shows the relevant structural requirements that acute hospitals must fulfill in order to carry out IER.

**Table 1:** Formal and structural requirements for in-hospital interdisciplinary early rehabilitation [10].

Interdisciplinary early rehabilitation complex treatment	
	may only be used as long as acute inpatient treatment is required
	operations and procedures code 8–559 in German diagnosis related group system (G-DRG)
	Not included: Geriatric early rehabilitation and neurological early rehabilitation
Structural requirements	
	Early rehabilitation team
	Rehabilitation specialist team leader (PRM specialist or at least 5 years experience in rehabilitation medicine)
	Nursing staff specially trained in activating-therapeutic care (therapeutic positioning, mobilization, activities of daily living, perceptual support, activation therapy, tracheal cannula management, etc.)
	At least 4 of the following therapies: Physiotherapy/physical therapy, occupational therapy, neuropsychology/psychology, psychotherapy, speech therapy/facio-oral therapy, art and/or music therapy, dysphagia therapy
	Standardized early rehabilitation assessment
	Weekly team meeting with goal setting and goal attainment scaling
	Structured discharge assessment

Infection with SARS-CoV-2 can cause a severe course of COVID-19 [11]. Sepsis and ARDS are common complications of a severe course [12]. Early physiotherapy and early mobilization for COVID-19 patients—not only in the intensive care unit (ICU)—have shown clinical benefits in several studies [7,13–15]. In severely affected patients, early physical therapy is associated with a reduced risk of prolonged ICU stays [14]. Therefore, some national clinical guidelines have adopted recommendations for early rehabilitative measures for these patients while they are still in the acute hospital [12,16–18]. In addition to the direct structural or organ damage caused by the infection, these cases often present immobility-related or therapy-related sequelae with functional impairments [7,19–21]. Although the data on the outcome of ER in this special patient group is still limited, several studies have already demonstrated positive effects on various functional parameters [16,22–24].

The German national *“Network University Medicine (NUM)”* was founded at the beginning of the COVID-19 pandemic with the aim of facilitating national cooperative research. A sub-project is the *“National Pandemic Cohort Network (NAPKON)”* with three different multi-center prospective observational cohorts. One was recruited across the health system sectors (SUEP), one at high resolution only in a university hospital context (HAP), and one population-based (POP) [25]. The SUEP cohort includes the documentation of IER in acute hospitals.

SUEP data are therefore suitable for investigating the aim of this study: the utilization of IER in German acute hospitals in COVID-19.

## Materials and methods

### Data sources

Since November 4, 2020, positive SARS-CoV-2 patients and controls without infection from all age groups have been recruited into the SUEP cohort in NAPKON at 57 locations in various areas of the healthcare system throughout Germany. These locations include university hospitals, non-university hospitals and general practitioners’ practices. Among demographic and clinical data, the SUEP database also records whether patients received in-hospital IER.

### Study design

IER utilization data was extracted from the SUEP cohort of hospitalized cases and stratified for prior intensive care treatment in a first step. For clinical analyses, a SUEP database export of the cases with an existing 3-month-follow-up (3MFU) was retrieved. Patients were included in this analysis if they were at least 18 years old, hospitalized for COVID-19 and had completed at least the 3MFU of the NAPKON-SUEP project. A descriptive analysis of this sample was carried out. Patients were characterized by age, height and weight, clinical frailty scale [26], and comorbidities. Cases with missing data in the 3MFU were not included in the data export.

Statistical analysis was performed using SPSS (version 28, IBM, Chicago, Illinois). Mean values and standard deviations were calculated for metric items as part of the descriptive analysis and to provide a better overview. Absolute frequencies and percentages were given for categorical and dichotomous variables. Proportions of utilization rates were calculated as the number of patients receiving the service divided by the total sample size. Corresponding 95% confidence intervals (CIs) were computed using the exact method by Clopper-Pearson based on the binomial distribution to ensure accurate interval estimation given the low event counts. Calculations were conducted using R (version 4.5.0, http://www.r-project.org).

Reporting was based on the STROBE guidelines for reporting cohort studies.

### Ethics and registration

For the NAPKON-SUEP cohort, a primary ethics vote was obtained from the ethics committee of the department of medicine at Goethe University Frankfurt, Germany (local ethics ID approval 20–924). All other study sites received local ethics votes from the respective ethics committees. Approval for this analysis was granted by the ethics committee of the medical faculty at the Friedrich Schiller University Jena, Germany (reference no. 2021–2493-Daten). The NAPKON-SUEP was registered with ClinicalTrials.gov (identifier: NCT04768998).

## Results

The sample was recruited between 12/01/2020 and 07/26/2023. The last 12MFU took place on 04/16/2024. The final data export from the SUEP database took place on 05/07/2024.

### IER utilization rates of hospitalized and ICU patients

**Fig 1:**
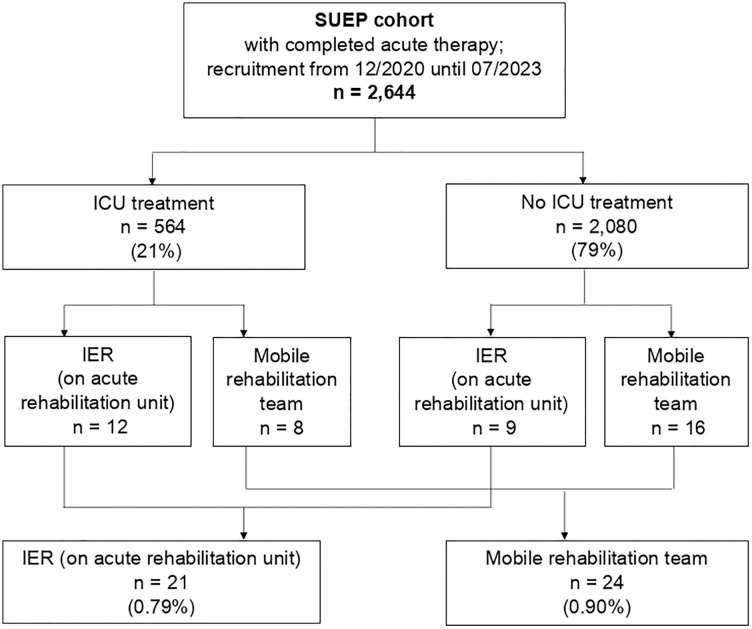
Flow chart. IER utilization data stratified by prior ICU treatment (SUEP: German abbreviation: Cross-Sectoral Platform, IER: in-hospital interdisciplinary early rehabilitation, ICU: intensive care unit).

The analysis of the entire database enabled an examination of IER utilization rates stratified by prior ICU treatment. At the time of data export, 2,644 patients of the entire SUEP cohort had completed acute inpatient treatment, of which 564 (21%) had received intensive care treatment during the course of their illness. IER on a specialized ward, i.e., acute rehabilitation unit (ARU), was documented for a total of n = 21 patients (0.79% [95% CI: 0.51%, 1.22%] of hospitalized cases), and therefore fulfilled the formal IER criteria. N = 24 patients (0.91% [95% CI: 0.60%–1.34%]) were visited by mobile rehabilitation teams, not fulfilling formal IER criteria. Fig 1 shows the detailed flow chart. Of the 564 patients in the SUEP cohort who had ICU treatment, 2.13% [95% CI: 1.16%, 3.63%] received in-hospital IER on an ARU.

### Clinical Analysis of ER Sample

**Fig 2:**
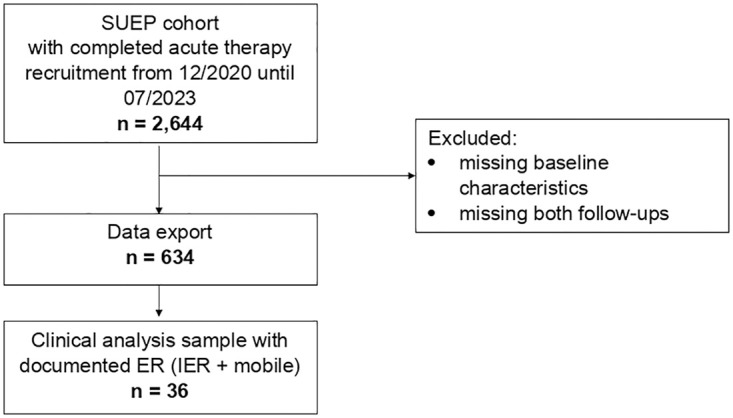
Flow chart. Clinical analysis sample with 3MFU (3MFU: 3 Month Follow Up, SUEP: German abbreviation: Cross-Sectoral Platform, ER: early rehabilitation, IER: in-hospital interdisciplinary early rehabilitation).

The data export with 3MFU data, selected according to the criteria described in the methods section, comprised 634 hospitalized patients with an existing 3MFU. A population of n = 36 (5.67% [95% CI: 4.09%, 7.80%]) patients received ER services, including IER as well as mobile early rehabilitation services (flow chart in Fig 2). They were on average 65.4 (± 16.1) years old and n = 13 (36.1%) stated that they were female.

When looking at the documented comorbidities of the 36 patients, cardiovascular diseases were predominantly documented (n = 24; 66.7%). This was followed—in descending order of frequency—by chronic neurological or psychiatric diseases (n = 13; 36.1%), chronic lung diseases (n = 10; 27.8%), and diabetes mellitus (n = 7; 19.4%). The characteristics of the sample are shown in Table 2.

**Table 2:** Characteristics of the interdisciplinary in-hospital early rehabilitation subcohort (IER and mobile rehabilitation team).

IER cases	36	
ICU^a^ treatment	16 (44.4%)	
age (years)	65.4 ± 16.1	
female	13 (36.1%)	
height (cm)	173.2 ± 9.8	
weight (kg)	83.1 ± 28.5	
current smoker (n = 29)	1 (3.6%)	
employment status	7	fully employed
	1	part-time
	2	minor or irregular
	21	not employed
	13	> of which retired
	*5*	*no information provided*
level of education	3	no vocational qualification
	12	vocational qualification
	3	master craftsman or technician
	2	state examination, master level, diploma
	2	doctorate
	*14*	*no information provided*
clinical frailty scale (≥ 65 years, n = 19)	3.0 ± 1.6 points	
	1	very fit
	6	fit
	4	managing well
	3	living with very mild frailty
	2	living with mild frailty
	3	living with moderate frailty
	*2*	*no information provided*
clinical frailty scale (< 65 years, n = 12)	2,0 ± 1,0 points	
	1	very fit
	7	fit
	3	managing well
	1	living with mild frailty
	3	*missing*
comorbidities (n)	4	none
	20	1-2
	10	3-5
	2	> 5
comorbidities (disease type)	24	cardiovascular (66.67%)
	13	neurological/psychiatric (36.11%)
	10	pulmonary (27.78%)
	7	rheumatological, immunological (19.44%)
	6	endocrinological (16.67%)
	5	solid tumor (13.89%)
	3	nephrological (8.33%)
	1	hepatic (2.78%)
death	3	
prone positioning	7	
oxygenation	23	oxygenation, of which:
	22	low-flow oxygen therapy
	10	high-flow oxygen therapy
	6	non-invasive ventilation
	10	invasive ventilation
	5	ECMO^b^

Values: mean ± standard deviation, percentages where indicated.

^a^ ICU: intensive care unit.

^b^ ECMO: extracorporeal membrane oxygenation.

## Discussion

### COVID-19 IER utilization rates compared with other data

This multicenter cohort study provides insight into the frequency of IER in acute hospitals for patients with COVID-19 in the NAPKON-SUEP cohort. The IER utilization rate in the acute hospital was 0.8% across the SUEP cohort and 2.1% among the subgroup of patients who received intensive care treatment during the course of their illness.

Previous scientific analyses on IER in Germany predominantly report clinical outcomes, while utilization rates are not precisely analyzed [6–8]. According to one estimate, the need for early rehabilitation therapy in acute hospitals is generally estimated between 1–3% of all acutely hospitalized patients [1].

In comparison, our observed COVID-19 sample shows a markedly lower utilization rate, suggesting a significant undersupply of IER. This interpretation is further supported by data from sepsis survivors in Germany, where the IER utilization rate is approximately 4.4% [5]. Notably, although this rate is more than five times higher than that observed in our COVID-19 cohort, it is still regarded as insufficient—highlighting an ongoing “unmet need” for IER, even in high-risk populations [5]. Drawing on findings from this interdisciplinary sepsis cohort, it can be hypothesized that early rehabilitation may help reduce long-term mortality—potentially extending up to 10 years—in patients with severe COVID-19 [5]. These comparisons emphasize the importance of incorporating early rehabilitation into acute hospital care for patients with significant functional impairments [5].

### ICU patients’ utilization rates

Across all inpatient cases in Germany, the overall ICU treatment rate was approximately 9% in the year 2022 [27]. By comparison, COVID-19 patients had more than double this ICU admission rate (20%) in the year 2020 in Germany, suggesting a greater overall rehabilitation need [28]. In the SUEP cohort, 21% of patients—closely matching the national figure—required intensive care, further underscoring the expected need for ER in this population [28]. Despite the potential limitations in data quality, the analysis supports the following conclusion: the presumed need for ER within the acute hospital setting was not met for many COVID-19 patients—particularly not for those with prior ICU treatment.

### Possible barriers to IER utilization

The utilization of IER services in acute care hospitals faced several barriers during the COVID-19 pandemic, some of which can be generalized, whereas others are specific to the German healthcare context. Especially during the pandemic, structural limitations like restricted bed capacity and staff shortages may have hindered the timely initiation of IER. The prioritization of critical cases often led to the reallocation of rehabilitation resources and personnel, reducing accessibility. In some cases, entire units were temporarily closed due to infection control measures. However, as data in the SUEP cohort were collected beyond the acute phase of the pandemic, these pandemic-specific disruptions cannot fully explain the under-utilization. Additional contributing factors may include patient-level factors, such as failure in to identify individuals eligible for IER and a consequent lack of referral. This may reflect knowledge or awareness deficits in clinicians, limitations in training, or inadequate resource availability.

### Health system barriers

IER in acute hospitals is increasingly acknowledged as an essential component of high-quality, integrated functioning-oriented patient care. Both the German Medical Association (Bundesärztekammer, BÄK) and the World Health Organization (WHO) advocate for the establishment of structured early rehabilitation services within acute care settings. A recent appeal by the German Medical Council entitled *High-performance medicine requires high-performance rehabilitation* explicitly calls for the implementation of early rehabilitative care at all acute hospitals treating patients with complex medical needs [29]. While sequential rehabilitation capacities—particularly in neurological and geriatric specialties—are well developed in Germany, there remains a significant structural gap in providing (interdisciplinary) early rehabilitation services directly within acute care hospitals.

The WHO’s 2023 *Resolution on Strengthening Rehabilitation in Health System*s formally acknowledges rehabilitation as an essential health service and calls on member states to embed rehabilitation into health system planning at all levels. The resolution highlights the importance of early initiation of rehabilitation interventions to improve patient outcomes and reduce disability. Complementing this, the WHO’s *Rehabilitation 2030* initiative provides detailed recommendations for implementing rehabilitation services, emphasizing timely, multidisciplinary, and patient-centered approaches starting in the acute phase. This also includes clear infrastructure development recommendations to implement specialized acute rehabilitation units: “Hospitals should include specialized rehabilitation units for inpatients with complex needs” [30].

These international frameworks underscore the urgent need to expand in-hospital IER capacities in Germany to ensure equitable access and optimize recovery trajectories for all patient populations—not only in the post-pandemic care for COVID-19 patients but also following other serious illnesses resulting in functional impairments [1].

Furthermore, it must be assumed that the documented mobile early rehabilitation teams in some hospitals do not meet the structural or therapeutic standards of dedicated acute rehabilitation units and instead provide services closer to early mobilization [29]. For this reason, such services were excluded from our utilization rate analysis.

ER provided sequentially after initial acute care—such as transfer to specialized neurological or geriatric rehabilitation hospitals or clinics—is likely not captured in the SUEP documentation. If included in an overall assessment, utilization proportions would potentially increase; however, this would not align with the definition of in-hospital IER as analyzed here.

Also, from a regulatory perspective, current reimbursement structures under the German Diagnosis-Related Groups (G-DRG) system may have be a barrier for implementing early rehabilitation services due to perceived financial constraints or inadequate reimbursement.

Taken together, two recurring structural weaknesses in the German early rehabilitation landscape can be identified: (1) the lack of integrated ER services within acute care hospitals, and (2) the limited availability of ER services for non-neurological, non-geriatric populations in the sequential rehabilitation phase following discharge from acute hospital care.

The opportunity presented by the forthcoming healthcare reforms should therefore be leveraged to address these structural deficits and enable the timely, i.e., earliest possible access to ER for patients with complex needs during their initial hospitalization.

### IER sample analysis

Among the 36 patients in the SUEP cohort who received IER, a majority of n = 22 patients reported having been very fit, averagely active or still coping well with everyday life prior to the SARS-CoV-2 infection—consistent with the Clinical Frailty Scale (CFS). However, it should be noted that the CFS is only validated for the age group over 64 years [34]. Among the 19 patients aged 65 or over, the distribution of responses was varied, although the most common (n = 6) premorbid functional scale was “managing well”. The scale was frequently used as a triage tool during the COVID-19 pandemic to make informed clinical decisions for further treatment when treatment resources were scarce [31]. The meta-analysis by Pranata et al. (2021) confirmed that an increasing CFS score is linearly associated with higher COVID-19 mortality [32]. Marti-Pastor et al. (2023) also showed that mild to moderate frailty was associated with increased readmission and also served as a predictor independent of disease severity [33,34]. In the cohort examined, pre-existing functional limitations were relatively minor and therefore likely did not negatively impact the intensity of subsequent treatment, potentially leading to lower IER needs and utilization.

### Limitations

While the analysis of the SUEP database promises robust data due to its sample size and multicenter design, there are major methodological limitations and analytical constraints. This analysis was only possible in the SUEP cohort of the NAPKON project. However, many patients who were treated at university hospitals, including hospitals with large IER units, were recruited into the high-resolution cohort (HAP), which is another NAPKON cohort. Unfortunately, no item concerning IER was included in the HAP database. For this reason, the actual utilization rate of IER services in hospital may differ from the results presented here, and a lack in generalizability of the results must be acknowledged.

It should also be mentioned that originally a matched-pairs design was planned to compare the quality of life of patients with and without IER at 3MFU and 12MFU. However, after an intensive review of the data export, it turned out that it contained incorrect data sets relating to other rehabilitation services. After the exclusion of data sets with missing data, the adjusted NAPKON export was then too small to analyze and the original comparative design had to be abandoned, which further weakens our study.

A relevant limitation of this analysis is certainly the small sample size, especially the low event count and consequently low utilization rates, which lead to leveraged bias. Therefore, further analyses beyond a descriptive presentation were not expedient.

## Conclusions

Early rehabilitation is an important part of acute medical treatment for severely affected patients and therefore also for COVID-19 patients. This study demonstrates a markedly low utilization of interdisciplinary early rehabilitation (IER) in acute care hospitals among COVID-19 patients in Germany, including those with prior ICU treatment. The findings suggest a substantial unmet need for in-hospital IER, likely driven by systemic and structural barriers rather than pandemic-specific factors alone. Strengthening IER capacities—particularly within acute care settings—should be prioritized to align with both the WHO resolution on rehabilitation at international level and the resolution of the German Medical Council at national level to ensure more equitable access to early functional recovery support.
